# High hip center bipolar hemiarthroplasty for non-reconstructable pelvic discontinuity: 3 hips in 2 patients

**DOI:** 10.3109/17453670902876797

**Published:** 2009-04-01

**Authors:** Byron E Chalidis, Michael D Ries

**Affiliations:** Department of Orthopaedic Surgery, University of CaliforniaSan Francisco, CAUSA

## Patient 1

**Figure 1.**
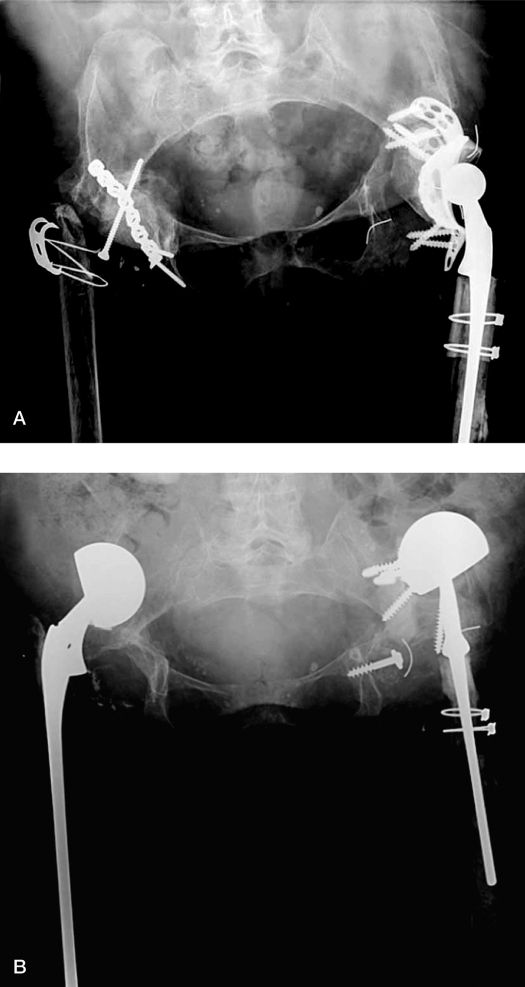
Patient 1. A. Bilateral pelvic discontinuity after failed treatment with structural allografting and cage fixation (left hip) and bone grafting with Girdlestone arthroplasty (right hip). B. Both hips were converted to bipolar hemiarthroplasty after several unsuccessful surgical attempts. No effort was made to further stabilize or reconstruct the bilateral pelvic discontinuities.

A 79-year-old woman with bilateral lower extremity weakness due to cervical myelopathy presented at our department in 2002 after multiple reconstructive procedures in both hips for developmental dysplasia of the hip. In 1993, a bulk allograft in combination with an acetabular cage and a cemented cup were used to treat the left massive acetabular bone loss. The defect was type IVb by the classification of the American Academy of Orthopaedic Surgeons (D'Antonio et al. 1989) and Berry et al. (1999). In 2000, the acetabular construct failed mechanically while the existing cemented femoral stem remained well fixed (Figure 1A). Removal of the acetabular hardware was followed by implantation of a whole acetabular allograft. The allograft was stabilized with plates and screws, and a new cemented cup was inserted. 2 years later, allograft fracture and acetabular failure occurred again.

In 2002, a high hip center bipolar hemiarthroplasty was performed via a standard posterior hip approach. Failed acetabular component and hardware were removed but the femoral stem was left in situ as it was found to be stable. Capsular and periacetabular scar tissues were preserved as much as possible to create a soft tissue cavity to seat the bipolar head into. A 60-mm bipolar femoral head was inserted onto the femoral component to articulate with the periacetabular soft tissues in a high hip center mode. Its position was further augmented with capsular repair around the neck of the prosthesis (capsular noose). A femoral condyle allograft was fixed to the ilium to serve as posterior superior acetabular wall. Postoperatively, the patient was advised to gradually increase her weight bearing using a walker or crutches. No casts or braces were applied. Within 6 months, the bipolar component migrated out of the acetabulum and articulated with the iliac soft tissues (Figure 1B). Although the patient had limb shortening, she had no pain and declined further surgery.

The right hip required 7 reconstructive procedures, which led to pelvic discontinuity (type IVb) and resection arthroplasty in 1999 (Figure 1A). In 2000, a re-implantation was performed by using a reinforcement ring with a cemented polyethylene cup and a long cemented femoral prosthesis. 4 years later the acetabular construct failed. A 56-mm diameter bipolar head was inserted onto the previously implanted and stable femoral stem and articulated with the soft tissues adjacent to the lateral ilium (Figure 1B). After surgery, the patient was able to transfer independently and ambulate short distances in her home with a walker.

At 3 years postoperatively (right hip) and 5 years postoperatively (left hip) the patient had no pain, relatively equal leg lengths, and could sit comfortably. Due to complete loss of lower extremity motor function associated with failed spine surgery and cervical myelopathy, the patient was non-ambulatory. However, the Harris hip score (HHS) of the patient’s left hip had improved from 39 preoperatively to 58 postoperatively. Similarly, the HHS of the patient’s right hip increased from 14 preoperatively to 58 postoperatively.

## Patient 2

**Figure 2.**
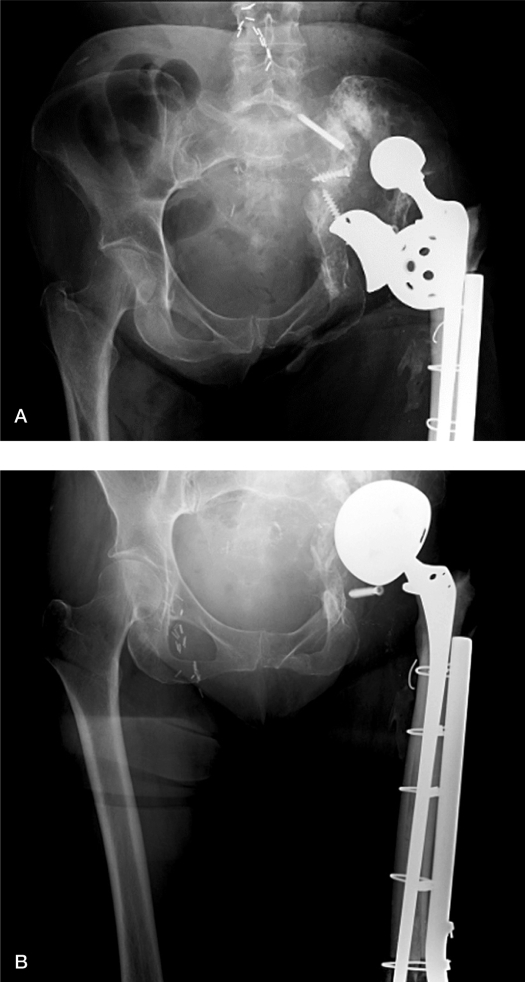
Patient 2. A. Non-reconstructable left pelvic discontinuity after failed treatment with a cementless cup and trabecular metal augments. The femur had been reconstructed with a segmental allograft prosthetic composite. B. Removal of the acetabular component was combined with insertion of a large bipolar head on the existing femoral prosthesis.

A 64-year-old woman with prior pelvic irradiation for non-Hodgkin’s lymphoma underwent total hip arthroplasty (THA) and 6 subsequent revision THAs for mechanical failure and recurrent hip dislocation. She presented with pelvic discontinuity (type IVc) pelvic osteonecrosis, and sciatic nerve palsy in 2002. This was treated using a bulk allograft fixed with posterior plating, a reinforcement ring, and a cemented cup. After 1 year, infection developed and a 2-stage revision arthroplasty was carried out. 2 years later, a periprosthetic femoral fracture occurred in association with loosening of the acetabular construct. The acetabulum was reconstructed with a composite of bulk allograft and hemispherical cementless cup supplemented with trabecular metal augments while the femoral component was revised to a total femur. Mechanical failure of the acetabular reconstruction occurred within 3 months (Figure 2A). A 70-mm diameter bipolar head was inserted onto the unrevised femoral stem and was stabilized by the soft tissues along the lateral ilium (Figure 2B).

At 1-year follow-up, the patient could transfer and ambulate independently using crutches and reported substantial relief of pain. The HHS was increased from 24 preoperatively to 51 postoperatively. The affected limb was 2 cm shorter than the contralateral side and a shoe lift was used to compensate for leg length inequality.

## Discussion

Large-bearing bipolar prosthesis with bone grafting has been used as a salvage procedure to treat recurrent hip dislocation and marked bone loss around failed cemented cups (Murray 1990, Roberson and Cohen 1990, Namba et al. 1994). Originally, this method was intended for temporary use as the first stage of a 2-stage revision while awaiting bone graft incorporation. As soon as adequate bone stock was achieved, a fixed type of THA was inserted. Some patients reported sufficient relief of pain that the second stage was omitted (Murray 1990). However, the outcome of the technique as a definitive procedure was considered to be poor due to motion between the outer bipolar surface and bone, which led to progressive bone loss and component migration. Eventually, the method was abandoned although it was considered superior to excision arthroplasty (Murray 1990, Roberson and Cohen 1990).

We have used the bipolar femoral head to articulate with the periacetabular soft tissues as an alternative to excision arthroplasty in patients with non-reconstructable acetabular discontinuity. Removal of femoral prostheses may cause significant damage to host bone, increase the surgical time and the overall operative burden, and delay rehabilitation. Furthermore, proximal femoral stump or trochanteric impingement against the pelvic wall may limit abduction and give pain (Petty and Goldsmith 1980, Ballard et al. 1995). Apart from pain, functional results are also unclear and highly variable in the literature, as patient satisfaction has ranged from 14% to 100% (Ballard et al. 1995).

In the cases presented here, the bipolar component does not articulate with the pelvis and weight-bearing forces would not be expected to be transmitted through the acetabular defect. This may have contributed to the satisfactory pain relief. However, hip function and mobility were not improved; the patients were minimally ambulatory and required the use of a walker or crutches. The inability to achieve weight-bearing capacity is most likely related to the lack of mechanical support for transferring loads from the hip to the axial skeleton. Since significant neurological, medical, or other orthopedic impairments had already compromised the mobility of our patients, the limited hip function associated with a chronically dislocated and migrated bipolar prosthesis does not appear to have affected their activity level further. Ultimately, the final outcome was characterized by pain relief, improvement in sitting ability, and high level of satisfaction—as demonstrated by the first patient who underwent a high hip center bipolar hemiarthroplasty and elected to have the same operation performed on her contralateral hip. We believe that this technique is a reasonable alternative to excision arthroplasty for sedentary and low-demand patients with non-reconstructable pelvic discontinuity.
